# Can Women Have It All: Marriage, Family, Career, and Good Health?

**DOI:** 10.1093/geroni/igaa057.1634

**Published:** 2020-12-16

**Authors:** Julene Cooney

**Affiliations:** Syracuse University, Manlius, New York, United States

## Abstract

Women in the U.S. have been earning more education than their male peers since the mid-1990’s. High power career positions that have historically been dominated by men are increasingly being filled by women. At the same time, since 2000, the U.S. workforce participation rate for women aged 25 – 55 has leveled off and begun to fall. Called the “mommy-wars” in the media, a heated debate has ensued between those women who feel that the role of mother should preempt seemingly selfish desires to engage in career work, while others firmly believe that it is possible to have it all; work, marriage and family. The purpose of this research project is to determine the health implications of each lifestyle for women later in life. Using the Health and Retirement Study, I compare self-rated health trajectories for the subset of women who are married, have children, and have attended college – those who are most likely to have the option to “opt-out” of the workforce for a stay-at-home mother lifestyle. Controlling for income and childhood health, and using growth curves and multivariate regression, preliminary results indicate that educated, married women who work full-time and raise children are the healthiest after age 50, while educated, married women who stay home with their children and do not work are the least healthy. In addition, these health trajectories have changed little since the 1950’s, even as cultural expectations for women have shifted.

